# Supplementary material for: A neuronal code for object representation and memory in the human amygdala and hippocampus
Source: Nat Commun. 2025 Feb 10;16:1510. doi: 10.1038/s41467-025-56793-y (PMC11811184; doi:10.1038/s41467-025-56793-y)
Supplement: Supplementary file 1 — Supplementary Information [file 41467_2025_56793_MOESM1_ESM.pdf]

Supplementary Information

Supplementary Results

Region-based feature coding with fixation-based analysis and natural scene stimuli

We have shown region-based feature coding above using isolated images in the central visual field. Do neurons also exhibit region-based feature coding when patients view natural scene images?

To answer this question, we employed a continuous recognition memory task (see **Methods**; **Supplementary Fig. 4a**) with 200 well-characterized natural scene images [1,2]. Patients indicated whether they had seen each image before on a trial-by-trial basis (accuracy = 79.67%±10.47% [mean±SD across sessions]). All faces and objects within these images have been manually segmented and delineated, leading to a total of 5551 semantic items; and we used ResNet to extract features from these semantic items and construct a visual feature space (**Supplementary Fig. 4b, c**). Here, we used the contents of fixations to study neural feature coding, which is much more efficient than viewing a single object at a time.

We recorded from neurons in the amygdala and hippocampus of 10 neurosurgical patients (2 male; 27 sessions in total). 684 neurons had an overall firing rate greater than 0.15 Hz and we restricted our analysis to this subset of neurons, which included 313 neurons from the amygdala, and 371 neurons from the hippocampus. Using the same selection procedure, we identified 73 (10.67%, binomial $P = 7.40\times10^{-10}$) feature neurons (31 from the learning session and 42 from the recognition session), with each feature neuron covering approximately 1.64%-8.55% of the feature space. Therefore, we have shown that region-based feature coding exists when we used natural scene stimuli with fixation-based analysis.

Memorability encoding in the recognition phase of the phased recognition memory task

In the recognition phase of the phased recognition memory task, we identified 113 neurons (9.72%, binomial $P = 1.43\times10^{-11}$; **Supplementary Fig. 5c, d**) that discriminated levels of image memorability (Pearson correlation between firing rate and memorability score, $P < 0.05$). Furthermore, we identified 140 feature neurons in the recognition phase. Compared to the whole population, feature neurons had a

significantly higher proportion of neurons that discriminated levels of image memorability (36/140, 25.71%, binomial $P < 10^{-20}$; χ^2-test between feature and whole: $P < 10^{-20}$). Notably, 72 out of 140 feature neurons had a significantly different memorability score between in-region and out-region stimuli (**Supplementary Fig. 5e**; 27 neurons significantly increased firing rate for in-region stimuli and 45 neurons significantly decreased firing rate for in-region stimuli). Similar to the learning phase (**Fig. 5**), we also found that at the population level in-region stimuli had a significantly lower memorability score compared to out-region stimuli (**Supplementary Fig. 5e**; two-tailed paired *t*-test, $t(139) = 2.97$, $P = 0.0035$, $d = 0.39$, 95% CI = [−0.04, −0.009]). Together, feature neurons are also associated with encoding image memorability during recognition.

Relationship between feature neurons and memory encoding neurons

In the phased memory recognition task, we identified feature neurons in both the learning phase (116/1162, 9.98%) and recognition phase (140/1162, 12.05%; χ^2-test between learning and recognition: $P = 0.11$; **Fig. 6a-h**). Consistent with our findings from the ImageNet and COCO stimuli, we observed that the majority of feature neurons in the learning phase (61/116, 52.59%) and recognition phase (78/140, 55.71%) exhibited category selectivity [3] (see **Fig. 6e, g** for examples). Furthermore, 13 (11.21%) feature neurons in the learning phase and 10 (7.14%) feature neurons in the recognition phase were memory-selective neurons (i.e., neurons coding novelty and familiarity; see [3,4] for definition). However, the overlap between feature neurons and memory-selective neurons was not higher than expected by chance (binomial test: Ps > 0.05), suggesting that these two populations of neurons were largely separate.

During the learning phase, among 104 neurons whose firing rate correlated with image memorability, 47 showed an increase in firing rate, while 57 exhibited a decrease in firing rate as a function of memorability. Furthermore, 31 of the feature neurons were memorability-encoding neurons, with the majority (27 neurons, 90%) showing a decrease in firing rate as a function of memorability (**Supplementary Fig. 5g**). At the population level, memorability had a negative modulatory effect on the response of feature neurons (**Supplementary Fig. 5f** left; two-tailed *t*-test against 0: $t(115) = 3.71$, $P = 3.16\times10^{-4}$, $d = 0.34$, 95% CI = [−0.08, −0.03]).

During the recognition phase, among 113 neurons whose firing rate correlated with image memorability, 62 showed an increase in firing rate, while 51 exhibited a decrease in firing rate as a function of memorability. Furthermore, 36 of the feature neurons were memorability-encoding neurons, with the majority (22 neurons, 61%) showing a decrease in firing rate as a function of memorability (**Supplementary Fig. 5h**). At the population level, we did not observe a significant modulation by memorability on the response of feature neurons (**Supplementary Fig. 5f** right; two-tailed *t*-test against 0: $t(139) = 1.58$, $P = 0.12$, $d = 0.13$, 95% CI = [−0.05, 0.006]). In addition, there was a comparable proportion of feature neurons encoding memorability, and the correlation was not significantly different between the learning phase and the recognition phase (**Supplementary Fig. 5f**; two-tailed two-sample *t*-test on Fisher *z*-transformed *r*: $t(254) = 1.52$, $P = 0.13$).

Memorability encoding in the continuous recognition memory task

We investigated whether feature neurons identified using fixation-based analysis and natural scene stimuli in the continuous recognition memory task (**Supplementary Fig. 4**) also encoded image memorability. The continuous recognition memory task had two sessions. In the learning session, 14 out of 31 feature neurons had significantly different memorability scores between in-region and out-region stimuli (2 neurons significantly increased firing rate for in-region stimuli and 12 neurons significantly decreased firing rate for in-region stimuli), suggesting that feature neurons' tuning regions differentiated image memorability. Consistent with the results derived from the phased recognition memory task (**Fig. 5**), at the population level, in-region stimuli had a significantly lower memorability score compared to out-region stimuli (two-tailed paired *t*-test: $t(30) = 3.21$, $P = 0.003$, $d = 0.79$, 95% CI = [−0.06, −0.01]). Notably, similar results were derived using a comprehensive saliency model [1] ($t(30) = 4.82$, $P = 3.90\times10^{-5}$, $d = 1.10$, 95% CI = [−0.05, −0.02]) as well as a Deep Gaze saliency model [5] ($t(30) = 4.47$, $P = 0.0001$, $d = 1.02$, 95% CI = [−0.07, −0.03]). In the recognition session, 19 out of 42 feature neurons had significantly different memorability scores between in-region and out-region stimuli (5 neurons significantly increased firing rate for in-region stimuli and 14 neurons significantly decreased firing rate for in-region stimuli). Although at the population level we did not observe a significant difference between in-region and out-region stimuli for memorability ($t(41) = 1.59$, $P = 0.12$, $d = 0.34$, 95% CI = [−0.04, −0.005]), we observed a significant difference for saliency (comprehensive saliency model [1]:

$t(41) = 1.98$, $P = 0.05$, $d = 0.41$, 95% CI = [−0.03, 0.0003]; Deep Gaze saliency model [5]: $t(41) = 2.74$, $P = 0.0092$, $d = 0.54$, 95% CI = [−0.05, −0.007]). Together, using fixation-based analysis with natural scene stimuli, we replicated our finding that feature neurons encoded image memorability.

Supplementary Discussion

Object space and DNN-based approaches to study neural object representation

To comprehensively investigate neural coding of objects, in this study, we employed an object space approach, historically used to study face [6,7] and object [8-13] representations in the brain. Object space is a metaphorical representation of how people perceive objects, where the axes represent perceptual features (e.g., color, shape) used to encode objects. Each object has a value on each feature axis, determining its position in the feature space. Object space has proven to be a valuable tool in numerous impactful studies of object perception [10,11,13], including special cases like faces [6,7,14-16].

Recent rapid advances in deep learning have provided an unprecedented opportunity to extract visual features from real-world natural images of objects and generate new unique objects by manipulating these features. This not only provides well-controlled stimuli to investigate differences in neural responses to feature changes (e.g., [17-19]), but also enables the use of more realistic, real-world stimuli to investigate object processing in the human brain. Inspired by the primate visual system, deep neural networks (DNNs) have made impressive progress on the complex problem of recognizing objects across variations of camera distance, viewpoint, and luminance. This powerful tool has been widely used to study object recognition and representation. For example, both human neuroimaging [20] and electrophysiology [21] studies have shown that features in DNNs can be represented in the human brain, which explains our ability to recognize individual faces (see [22] for simulation work). Recent studies in monkeys have shown that images synthesized by DNNs can control neural population activity [18,19]. It has also been shown that unsupervised deep learning identifies semantic disentanglement in single inferotemporal neurons [23], and the geometry of the object manifolds has been illustrated in DNNs [12]. Notably, using natural face stimuli and face features extracted from DNNs, our recent work has shown that neurons in the human amygdala and hippocampus exhibit region-based feature coding and encode visually similar identities [24]. Together, these recent studies have demonstrated compelling evidence that DNNs are appropriate and useful tools to study neural object coding.

Possible caveats

All patients have a diagnosis of intractable epilepsy. While it is unrealistic to entirely discount the possibility that our findings are affected by the disease, we observed feature neurons in the "healthy" tissue (defined as outside the seizure onset zone). Furthermore, while the amygdala and hippocampus are crucial components of the object processing network, our current study was restricted by clinical limitations, which prevented us from examining the broader neural circuitry involved in object processing. Given the distributed nature of object processing in the temporal cortex [11] and the diverse functions of different subregions of the human medial temporal lobe (MTL) [25-28], future studies should explore neural object coding at the network level. Additionally, although we found that the amygdala and hippocampus showed qualitatively similar feature coding (**Supplementary Fig. 2**), different nuclei of the amygdala and various subregions of the hippocampus may play different roles in object coding and memory. Therefore, a future study is needed to systematically investigate the functional roles of amygdala and hippocampal subregions.

It is worth noting that more than one DNN model can be used to study feature coding because different sets of visual features can be employed for object recognition. In our previous study [24], we conducted systematic control analyses to explore the specificity of the feature space and stimuli in identifying feature neurons. We found that face feature neurons could be identified using other face recognition DNNs, even in feature spaces constructed using DNNs trained for object recognition. This similarity was likely due to the similarity between DNN models [24,29]. However, on the other hand, we also found that MTL neurons were sensitive to the organization of the feature space, and thus, the organization of the feature space played a critical role in identifying feature neurons. Similarly, in the present study, we found that both ResNet and AlexNet, as well as different layers of these networks, can be used to identify feature neurons. In addition, although we selected feature neurons in a two-dimensional feature space (after reducing the full features to two dimensions), we could replicate our findings using the full DNN features. It is worth noting that some objects within the region did not elicit an elevated response compared to objects outside the region because their features were not separable in the two-dimensional feature space; rather, they were only separable using the full features.

Lastly, we acknowledge that in our recognition memory tasks, the classification of remembered vs. forgotten items is inherently behavior-driven rather than experimentally manipulated, which may introduce potential confounds such as attentional lapses or patient-specific factors. We also recognize that our study focuses on a specific subset of MTL neurons and provides correlational rather than causal

evidence for the proposed response model. Nonetheless, our findings represent an important initial step toward understanding how MTL neurons transform visual features into memories, highlighting a critical link between visual feature coding and memory formation. We believe our work will inspire further research to explore these mechanisms more comprehensively using causal methodologies.

ID	Sex	Age	Race	Epilepsy diagnosis	Number of Amygdala Neurons				Number of Hippocampus Neurons			
					Total	Left	Right	Feature	Total	Left	Right	Feature
WVU14	M	26	Caucasian	Bilateral amygdala and hippocampus	16	12	4	2	9	4	5	0
WVU16	F	29	Caucasian	Right temporal neocortex	34	18	16	2	37	24	13	2
					38	24	14	4	47	25	22	2
WVU18	F	53	Caucasian	Left temporal	7	7	0	0	1	0	1	0
					54	26	28	1	8	3	5	1
					30	14	16	1	6	2	4	1
WVU19	M	49	Caucasian	Right mesial temporal	1	0	1	0	18	10	8	0
					1	0	1	1	25	11	14	5
WVU20	F	31	Caucasian	Left temporal	38	34	4	9	52	52	0	9
					34	31	3	5	25	25	0	1
					34	31	3	8	27	27	0	0
					36	34	2	3	25	25	0	0
WVU21	F	27	Caucasian	Left mesial temporal	31	31	0	0	0	0	0	0
					32	32	0	2	0	0	0	0
UIC202214	F	45	Caucasian	Right mesial temporal	10	10	0	5	7	1	6	1
BJH024	F	38	Caucasian	Left temporal	13	0	13	0	8	0	8	0
BJH025	F	45	Caucasian	Right mesial temporal	9	9	0	0	0	0	0	0
BJH026	M	40	Caucasian	Left mesial temporal	19	19	0	3	13	13	13	0
					27	27	0	4	11	11	11	0
BJH027	M	37	Caucasian	Right temporal	0	0	0	0	18	18	0	18
					0	0	0	0	14	14	0	14
BJH028	F	36	Caucasian	Left frontal SMA, cingulate regions	21	21	0	7	0	0	0	0
BJH029	F	34	Caucasian	Left amygdala and hippocampus	0	0	0	0	8	8	0	8
					0	0	0	0	5	5	0	5
BJH032	M	27	Caucasian/ American Indian	Bilateral amygdala and hippocampus	0	0	0	0	6	0	0	0
					0	0	0	0	4	0	0	0
BJH033	F	24	Caucasian/ American Indian	Right hippocampus	11	11	0	3	1	1	1	0
					3	3	0	0	0	0	0	0
Sum					499	394	105	60	375	279	109	169

Supplementary Table 1. List of patients for the one-back task with the ImageNet stimuli. Each row of neurons represents a separate recording session using the ImageNet stimuli. Each session was recorded on a separate day. Total: all neurons recorded from an area. Left: neurons that were recorded from the

left side of an area and had a firing rate greater than 0.15 Hz. Right: neurons that were recorded from the right side of an area and had a firing rate greater than 0.15 Hz. These neurons were included for further analysis. Feature: feature neurons.

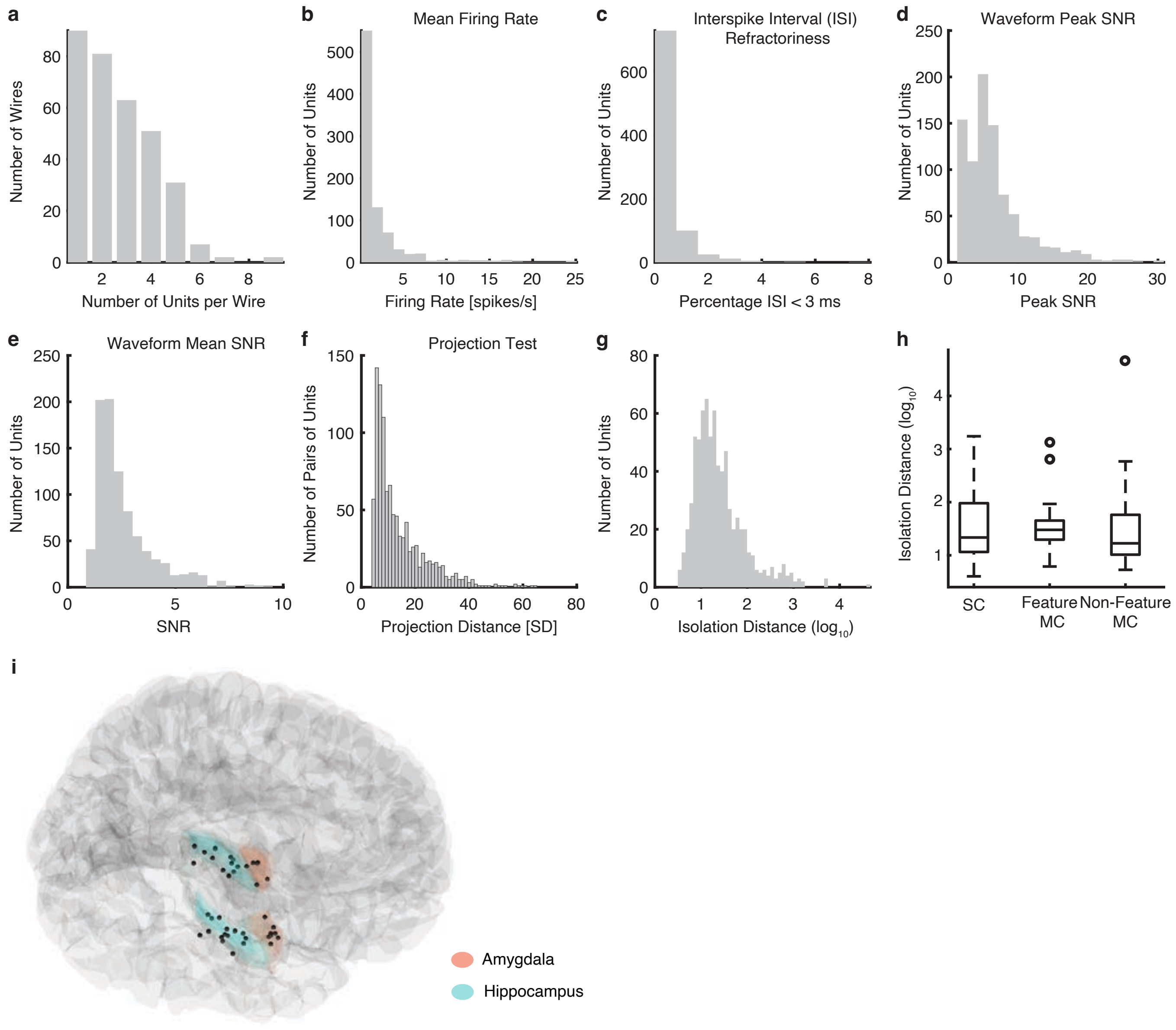

Supplementary Fig. 1. Spike sorting and recording quality assessment. **(a)** Histogram of the number of units identified on each active wire (only wires with at least one unit identified are counted). The average yield per wire with at least one unit was 2.67±1.51 (mean±SD). **(b)** Histogram of mean firing rates. **(c)** Histogram of proportion of inter-spike intervals (ISIs) which are shorter than 3 ms. The large majority of clusters had less than 0.5% of such short ISIs. **(d)** Histogram of the signal-to-noise ratio (SNR) of the mean waveform peak of each unit. **(e)** Histogram of the SNR of the entire waveform of all units. **(f)** Pairwise distance between all possible pairs of units on all wires where more than 1 cluster was isolated. Distances are expressed in units of standard deviation (SD) after normalizing the data such that the distribution of waveforms around their mean is equal to 1. **(g)** Isolation distance of all units for which this metric was defined (n = 874, median = 18.88). Isolation distance was calculated based on

[30,31]. If a cluster contains n_C cluster spikes, the isolation distance of the cluster is the D^2 value of the n_C th closest noise spike. Isolation distance is therefore the radius of the smallest ellipsoid from the cluster center containing all of the cluster spikes and an equal number of noise spikes. As such, isolation distance estimates how distant the cluster spikes are from the other spikes recorded on the same electrode. Isolation distance is not defined for cases in which the number of cluster spikes is greater than the number of noise spikes. **(h)** Single-category (SC), feature multiple-category (MC), and non-feature MC neurons did not differ significantly in isolation distance (one-way ANOVA: $F(2, 94) = 0.04$, $P = 0.96$). On each box, the central mark is the median, the edges of the box are the 25th and 75th percentiles, the whiskers extend to the most extreme data points the algorithm considers to be not outliers, and the outliers are plotted individually. n.s.: not significant. **(i)** Electrode locations. The recording sites of each patient (block dots) were estimated according to the pre-implantation T1-weighted MRI and post-implantation CT. Source data are provided as a Source Data file.

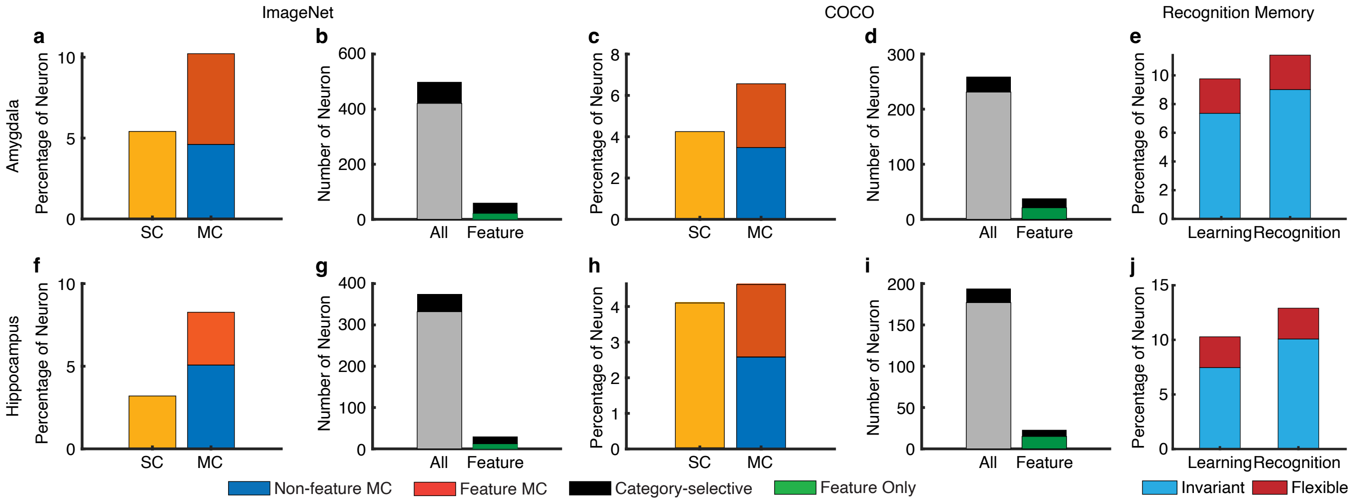

Supplementary Fig. 2. Population summary of neurons broken down for the amygdala and hippocampus. **(a-e)** Amygdala neurons. **(f-j)** Hippocampal neurons. **(a, b, f, g)** One-back task with the ImageNet stimuli. **(c, d, h, i)** One-back task with the Microsoft COCO stimuli. **(e, j)** Phased recognition memory task. **(a, c, f, h)** Percentage of single-category (SC; shown in yellow) and multiple-category (MC) neurons in the neuronal population. Stacked bar shows MC neurons that encoded visually similar objects (i.e., feature MC neurons; red) or not (i.e., non-feature MC neurons; blue). **(b, d, g, i)** The number of category-selective neurons in the whole population (left) and among feature neurons (right). Black: the number of category-selective neurons. Green: the number of non-category-selective feature neurons. Gray: the number of non-category-selective neurons. **(e, j)** Percentage of invariant (blue) and flexible (red) feature neurons in the learning and recognition task phases. Amygdala and hippocampal neurons showed a similar selectivity across tasks. Source data are provided as a Source Data file.

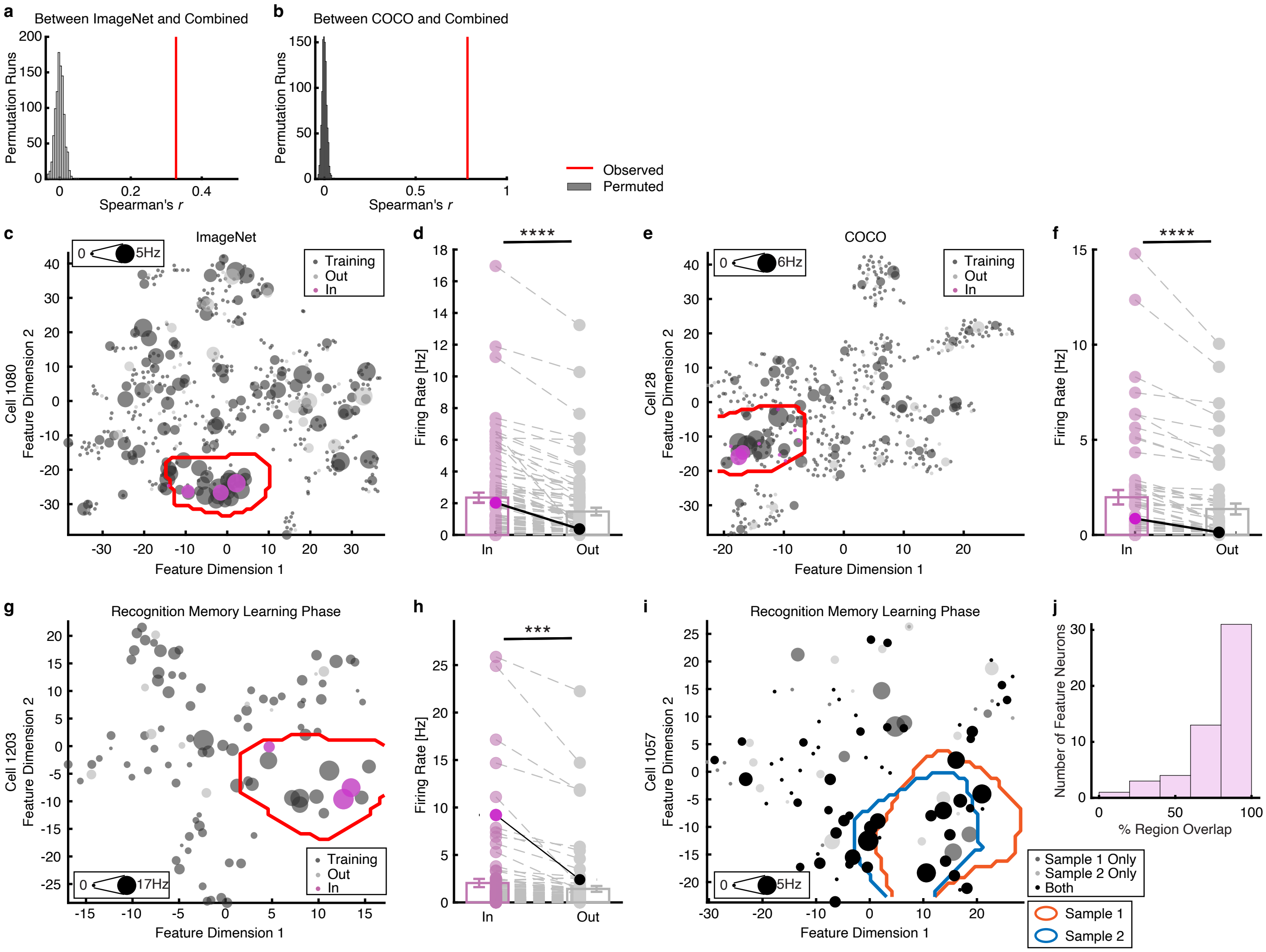

Supplementary Fig. 3. Control analyses for region-based feature coding. **(a, b)** Stability of the feature space across different constructions. We conducted a representational similarity analysis (RSA) between an individual feature space and the combined feature space. Stimulus-by-stimulus representational dissimilarity matrix (RDM) was calculated using stimulus coordinates from an individual feature space or the combined feature space. Using a permutation test, we found that the stimulus representations (i.e., stimulus coordinates in the feature space) in both the ImageNet ($P < 0.001$) and COCO ($P < 0.001$) feature spaces were significantly correlated with the stimulus representations in the combined feature space, suggesting that stimulus representations remained stable across different constructions of feature spaces and that the feature spaces were consistent across different image datasets. **(a)** ImageNet. **(b)** COCO. **(c-h)** Control analyses with training and testing stimuli from the same dataset. Here, we used an 8-fold cross-validation procedure within the ImageNet and COCO datasets. Specifically, we split the data into 8 folds, using 7 of them to select the tuning region, and the remaining fold to test the response within vs. outside the region. This procedure was repeated 8 times, and all the feature neurons selected

from the entire dataset were identified. We then performed the In vs. Out comparison on these feature neurons. We observed a significantly higher response to in-region stimuli compared to out-region stimuli in the ImageNet dataset (right-tailed paired *t*-test: $t(88) = 6.81$, $P = 5.76\times10^{-10}$), the COCO dataset ($t(60) = 5.34$, $P = 7.48\times10^{-7}$), and during the learning phase of the phased recognition memory task ($t(101) = 3.59$, $P = 2.56\times10^{-4}$). These results served as a positive control for cross-dataset generalization and cross-phase consistency. **(c, d)** ImageNet dataset. **(e, f)** COCO dataset. **(g, h)** Learning phase of the phased recognition memory task. **(d, f, h)** Population results comparing neuronal response to in-region testing stimuli vs. out-region testing stimuli. Legend conventions as in **Fig. 3c, d**. ***: $P < 0.001$, and ****: $P < 0.0001$. **(i, j)** Consistency of the coding regions during the learning phase of the phased recognition memory task. We randomly subsampled 80% of the data from the learning phase twice and compared the distribution of the identified tuning regions. **(i)** An example neuron. **(j)** Group summary. Legend conventions as in **Fig. 6e**. Source data are provided as a Source Data file.

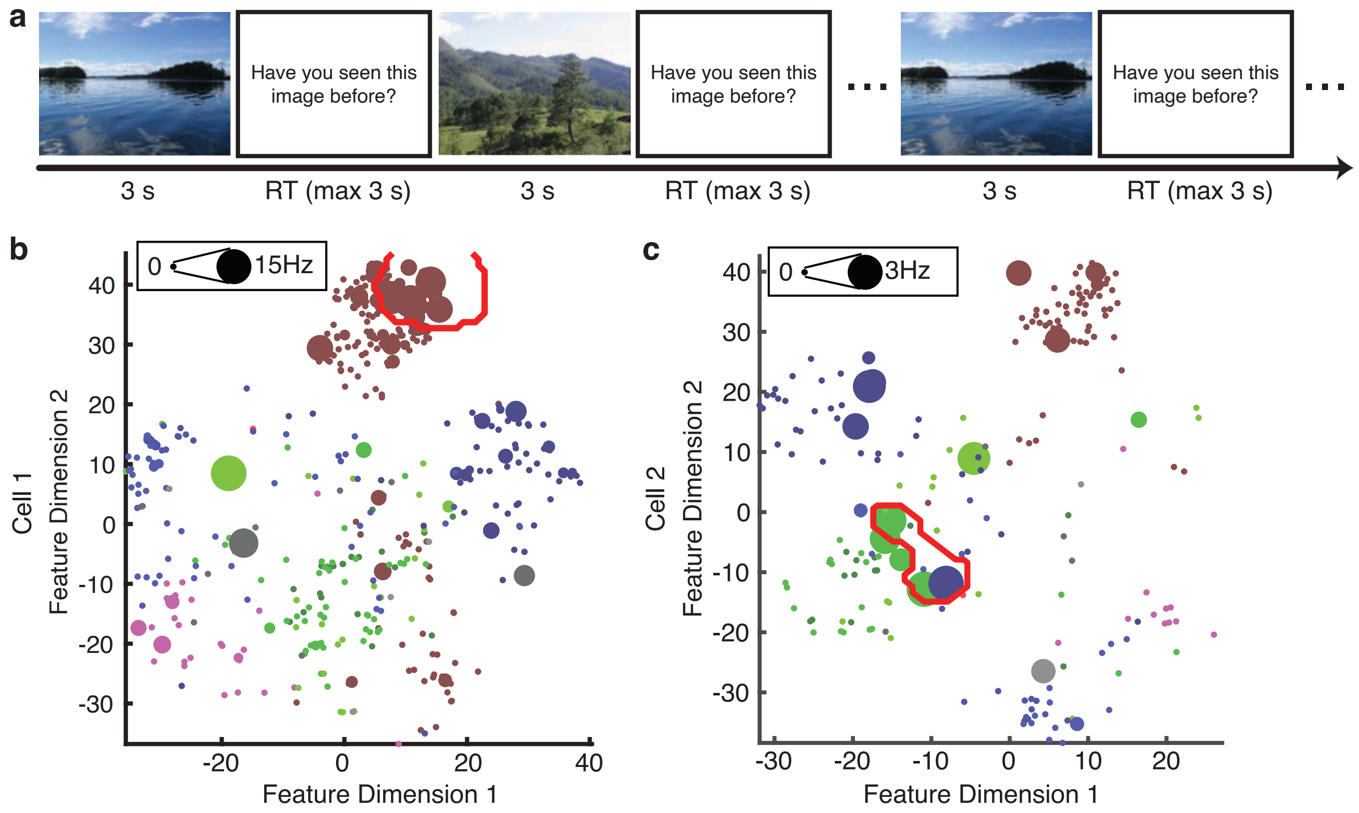

Supplementary Fig. 4. Validation of region-based feature coding using natural scene stimuli and fixation-based analysis. **(a)** Participants viewed each image for 3 s. After viewing each image, participants were asked, "Have you seen this image before?" and they were required to respond as soon as possible using a six-point scale (no sure, no less sure, no unsure, yes unsure, yes less sure, yes sure). Both the learning session and the recognition session involved an identical task. In the learning session, 100 unique images were selected, and 50 of these images were repeated. Therefore, the learning session consisted of a total of 150 trials. In the recognition session, another set of 100 images was selected, comprising the "new" stimuli. All 100 images from the learning session were also shown in the recognition session, comprising the "old" stimuli. The images used in the experiment were obtained from the OSIE dataset [1], while the images shown here for illustration purposes were obtained from Faraut, Mailys C. M.; Carlson, April A.; Sullivan, Shannon, et al. (2019). Data from: Dataset of human medial temporal lobe single-neuron activity during declarative memory encoding and recognition [Dataset]. Dryad. https://doi.org/10.5061/dryad.46st5. **(b, c)** Example neurons demonstrating region-based feature coding. Legend conventions as in **Fig. 1**. Source data are provided as a Source Data file.

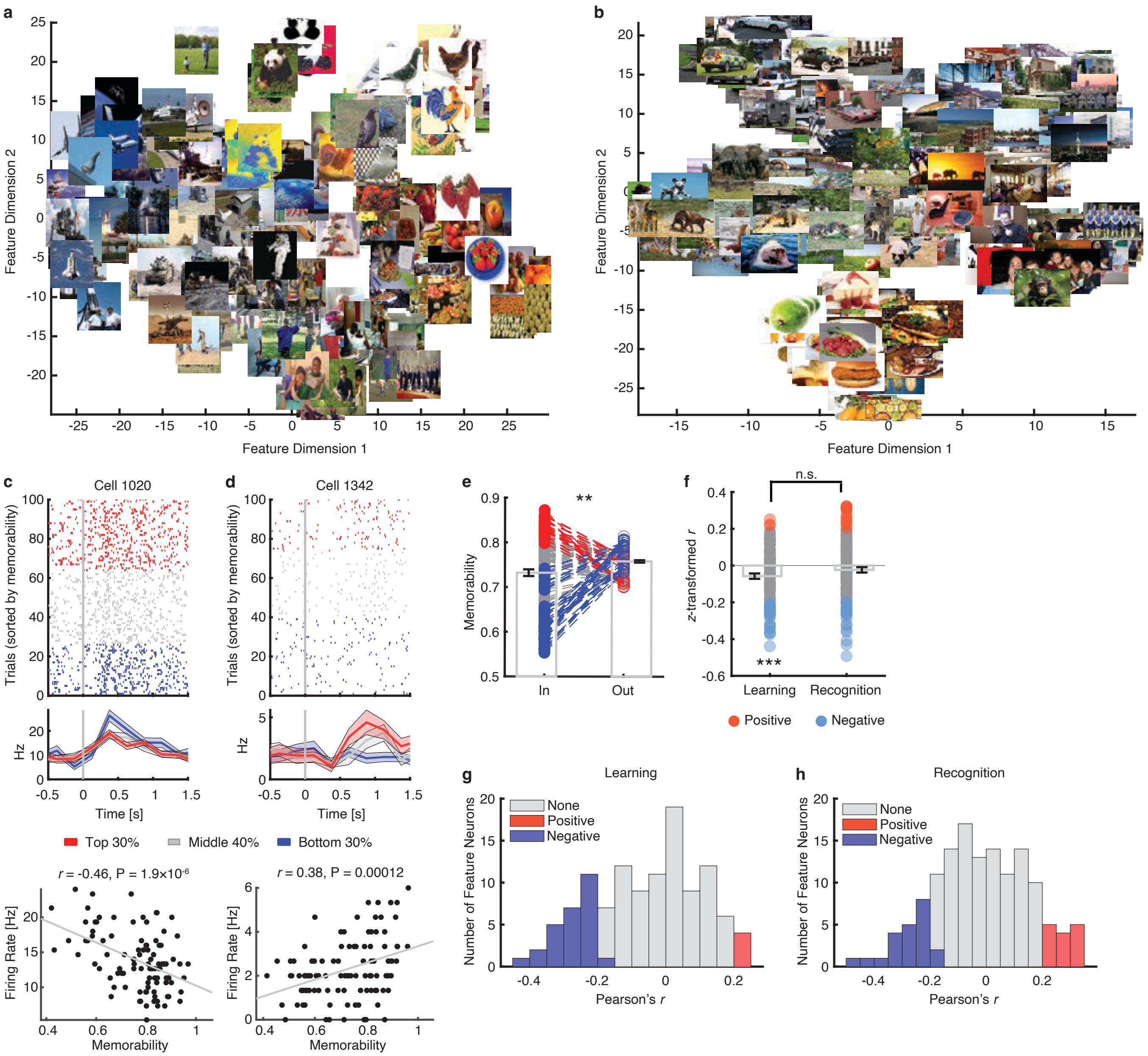

Supplementary Fig. 5. Additional results for the phased recognition memory task. **(a, b)** Example feature spaces constructed using stimuli from the phased recognition memory task (see **Methods**). **(a)** Stimuli from Task Variant 2. **(b)** Stimuli from Task Variant 3. Images are from Faraut, Mailys C. M.; Carlson, April A.; Sullivan, Shannon, et al. (2019). Data from: Dataset of human medial temporal lobe single-neuron activity during declarative memory encoding and recognition [Dataset]. Dryad. https://doi.org/10.5061/dryad.46st5. **(c, d)** Example neurons that differentiated levels of image memorability. Trials are aligned to the stimulus onset. Shaded area denotes ±SEM across trials. The firing rate of these example neurons was correlated with image memorability scores. Each dot represents an image, and the gray line shows the linear fit. **(e)** Memorability between in-region vs. out-region stimuli. Each circle

represents a feature neuron, and error bars denote ±SEM across all feature neurons. Red circles denote feature neurons that had a significantly higher response for in-region stimuli. Blue circles denote feature neurons that had a significantly higher response for out-region stimuli. Asterisks indicate a significant difference between in-region vs. out-region stimuli across all feature neurons using a two-tailed paired *t*-test. **: $P < 0.01$. **(f)** *z*-transformed correlation coefficient. Each circle represents a feature neuron, and error bars denote ±SEM across all feature neurons. Red circles denote feature neurons whose firing rate significantly positively correlated with memorability. Blue circles denote feature neurons whose firing rate significantly negatively correlated with memorability. Asterisks indicate a significant difference against 0 using a two-tailed paired *t*-test. ***: $P < 0.001$. There was no significant difference between the learning and recognition phases (two-tailed two-sample *t*-test). n.s.: not significant. **(g)** Distribution of correlation coefficient for the learning phase. **(h)** Distribution of correlation coefficient for the recognition phase. Pearson correlation was calculated between image memorability and each neuron's firing rate. Red: feature neurons whose firing rate significantly positively correlated with memorability. Blue: feature neurons whose firing rate significantly negatively correlated with memorability. Gray: feature neurons whose firing rate did not significantly correlate with memorability. Source data are provided as a Source Data file.

Supplementary References

1 Xu, J., Jiang, M., Wang, S., Kankanhalli, M. S. & Zhao, Q. Predicting human gaze beyond pixels. *Journal of Vision* **14**, 28 (2014). https://doi.org/10.1167/14.1.28

2 Wang, S. *et al.* Atypical Visual Saliency in Autism Spectrum Disorder Quantified through Model-Based Eye Tracking. *Neuron* **88**, 604-616 (2015). https://doi.org/http://dx.doi.org/10.1016/j.neuron.2015.09.042

3 Faraut, M. C. M. *et al.* Dataset of human medial temporal lobe single neuron activity during declarative memory encoding and recognition. *Scientific Data* **5**, 180010 (2018). https://doi.org/10.1038/sdata.2018.10

4 Rutishauser, U., Mamelak, A. N. & Schuman, E. M. Single-Trial Learning of Novel Stimuli by Individual Neurons of the Human Hippocampus-Amygdala Complex. *Neuron* **49**, 805-813 (2006). https://doi.org/10.1016/j.neuron.2006.02.015

5 Kümmerer, M., Theis, L. & Bethge, M. (2015).

6 Leopold, D. A., Bondar, I. V. & Giese, M. A. Norm-based face encoding by single neurons in the monkey inferotemporal cortex. *Nature* **442**, 572-575 (2006). https://doi.org/http://www.nature.com/nature/journal/v442/n7102/suppinfo/nature04951_S1.html

7 Chang, L. & Tsao, D. Y. The Code for Facial Identity in the Primate Brain. *Cell* **169**, 1013-1028.e1014 (2017). https://doi.org/10.1016/j.cell.2017.05.011

8 Mel, B. W. SEEMORE: Combining Color, Shape, and Texture Histogramming in a Neurally Inspired Approach to Visual Object Recognition. *Neural Computation* **9**, 777-804 (1997). https://doi.org/10.1162/neco.1997.9.4.777

9 Palmeri, T. J. & Gauthier, I. Visual object understanding. *Nature Reviews Neuroscience* **5**, 291-303 (2004). https://doi.org/10.1038/nrn1364

10 Cichy, R. M., Pantazis, D. & Oliva, A. Resolving human object recognition in space and time. *Nat Neurosci* **17**, 455-462 (2014). https://doi.org/10.1038/nn.3635 http://www.nature.com/neuro/journal/v17/n3/abs/nn.3635.html#supplementary-information

11 Bao, P., She, L., McGill, M. & Tsao, D. Y. A map of object space in primate inferotemporal cortex. *Nature* **583**, 103-108 (2020). https://doi.org/10.1038/s41586-020-2350-5

12 Cohen, U., Chung, S., Lee, D. D. & Sompolinsky, H. Separability and geometry of object manifolds in deep neural networks. *Nature Communications* **11**, 746 (2020). https://doi.org/10.1038/s41467-020-14578-5

13 Hebart, M. N., Zheng, C. Y., Pereira, F. & Baker, C. I. Revealing the multidimensional mental representations of natural objects underlying human similarity judgements. *Nature Human Behaviour* **4**, 1173-1185 (2020). https://doi.org/10.1038/s41562-020-00951-3

14 Leopold, D. A., O'Toole, A. J., Vetter, T. & Blanz, V. Prototype-referenced shape encoding revealed by high-level aftereffects. *Nat Neurosci* **4**, 89-94 (2001).

15 Loffler, G., Yourganov, G., Wilkinson, F. & Wilson, H. R. fMRI evidence for the neural representation of faces. *Nat Neurosci* **8**, 1386-1391 (2005).

16 Freiwald, W. A., Tsao, D. Y. & Livingstone, M. S. A face feature space in the macaque temporal lobe. *Nat Neurosci* **12**, 1187-1196 (2009). https://doi.org/http://www.nature.com/neuro/journal/v12/n9/suppinfo/nn.2363_S1.html

17 Yamins, D. L. K. *et al.* Performance-optimized hierarchical models predict neural responses in higher visual cortex. *Proceedings of the National Academy of Sciences* **111**, 8619 (2014). https://doi.org/10.1073/pnas.1403112111

18 Bashivan, P., Kar, K. & DiCarlo, J. J. Neural population control via deep image synthesis. *Science* **364**, eaav9436 (2019). https://doi.org/10.1126/science.aav9436
19 Ponce, C. R. *et al.* Evolving Images for Visual Neurons Using a Deep Generative Network Reveals Coding Principles and Neuronal Preferences. *Cell* **177**, 999-1009.e1010 (2019). https://doi.org/10.1016/j.cell.2019.04.005
20 VanRullen, R. & Reddy, L. Reconstructing faces from fMRI patterns using deep generative neural networks. *Communications Biology* **2**, 193 (2019). https://doi.org/10.1038/s42003-019-0438-y
21 Grossman, S. *et al.* Convergent evolution of face spaces across human face-selective neuronal groups and deep convolutional networks. *Nature Communications* **10**, 4934 (2019). https://doi.org/10.1038/s41467-019-12623-6
22 Wang, J., Cao, R., Brandmeir, N. J., Li, X. & Wang, S. Face identity coding in the deep neural network and primate brain. *Communications Biology* **5**, 611 (2022). https://doi.org/10.1038/s42003-022-03557-9
23 Higgins, I. *et al.* Unsupervised deep learning identifies semantic disentanglement in single inferotemporal face patch neurons. *Nature Communications* **12**, 6456 (2021). https://doi.org/10.1038/s41467-021-26751-5
24 Cao, R. *et al.* Feature-based encoding of face identity by single neurons in the human medial temporal lobe. *bioRxiv*, 2020.2009.2001.278283 (2020). https://doi.org/10.1101/2020.09.01.278283
25 Squire, L. R., Stark, C. E. L. & Clark, R. E. The Medial Temporal Lobe. *Annual Review of Neuroscience* **27**, 279-306 (2004). https://doi.org/10.1146/annurev.neuro.27.070203.144130
26 Bussey, T. J., Saksida, L. M. & Murray, E. A. The Perceptual-Mnemonic/Feature Conjunction Model of Perirhinal Cortex Function. *The Quarterly Journal of Experimental Psychology Section B* **58**, 269-282 (2005). https://doi.org/10.1080/02724990544000004
27 Eichenbaum, H., Yonelinas, A. P. & Ranganath, C. The Medial Temporal Lobe and Recognition Memory. *Annual Review of Neuroscience* **30**, 123-152 (2007). https://doi.org/10.1146/annurev.neuro.30.051606.094328
28 Murray, E. A., Bussey, T. J. & Saksida, L. M. Visual Perception and Memory: A New View of Medial Temporal Lobe Function in Primates and Rodents. *Annual Review of Neuroscience* **30**, 99-122 (2007). https://doi.org/10.1146/annurev.neuro.29.051605.113046
29 Keles, U., Lin, C. & Adolphs, R. A Cautionary Note on Predicting Social Judgments from Faces with Deep Neural Networks. *Affective Science* (2021). https://doi.org/10.1007/s42761-021-00075-5
30 Harris, K. D., Hirase, H., Leinekugel, X., Henze, D. A. & Buzsáki, G. Temporal Interaction between Single Spikes and Complex Spike Bursts in Hippocampal Pyramidal Cells. *Neuron* **32**, 141-149 (2001). https://doi.org/https://doi.org/10.1016/S0896-6273(01)00447-0
31 Schmitzer-Torbert, N., Jackson, J., Henze, D., Harris, K. & Redish, A. D. Quantitative measures of cluster quality for use in extracellular recordings. *Neuroscience* **131**, 1-11 (2005). https://doi.org/https://doi.org/10.1016/j.neuroscience.2004.09.066
